# Efficacy of Prophylactic Mesh in End-Colostomy Construction: A Systematic Review and Meta-analysis of Randomized Controlled Trials

**DOI:** 10.1007/s00268-016-3576-0

**Published:** 2016-05-23

**Authors:** Shuanhu Wang, Wenbin Wang, Bing Zhu, Guolei Song, Congqiao Jiang

**Affiliations:** 1 Department of Gastrointestinal Surgery, The First Affiliated Hospital of Bengbu Medical College, Bengbu, Anhui China; 2 Department of General Surgery, The Fourth Affiliated Hospital of Anhui Medical University, Hefei, Anhui China

## Abstract

**Background:**

Parastomal hernia is a very common complication after colostomy, especially end-colostomy. It is unclear whether prophylactic placement of mesh at the time of stoma formation could prevent parastomal hernia formation after surgery for rectal cancer. A systematic review and meta-analysis were conducted to evaluate the efficacy of prophylactic mesh in end-colostomy construction.

**Methods:**

PubMed, Embase, and the Cochrane Library were searched, covering records entered from their inception to September 2015. Randomized controlled trials (RCTs) comparing stoma with mesh to stoma without mesh after surgery for rectal cancer were included. The primary outcome was the incidence of parastomal hernia. Pooled risk ratios (RR) with 95 % confidence intervals (CI) were obtained using random effects models.

**Results:**

Six RCTs containing 309 patients were included. Parastomal hernia occurred in 24.4 % (38 of 156) of patients with mesh and 50.3 % (77 of 153) of patients without mesh. Meta-analysis showed a lower incidence of parastomal hernia (RR, 0.42; 95 % CI 0.22–0.82) and reoperation related to parastomal hernia (RR, 0.23; 95 % CI 0.06–0.89) in patients with mesh. Stoma-related morbidity was similar between mesh group and non-mesh group (RR, 0.65; 95 % CI 0.33–1.30).

**Conclusions:**

Prophylactic placement of a mesh at the time of a stoma formation seems to be associated with a significant reduction in the incidence of parastomal hernia and reoperation related to parastomal hernia after surgery for rectal cancer, but not the rate of stoma-related morbidity. However, the results should be interpreted with caution because of the heterogeneity among the studies.

## Introduction

Colorectal cancer is the third most common malignancy worldwide, but its mortality is considerably lower than that of other cancers [1]. Although surgical techniques and instruments have been greatly improved, many patients with rectal cancer still suffer permanent colon stoma. Parastomal hernia is a common complication of end-colostomy. The incidence of parastomal hernia varies from 16 to 57 % [2, 3]. Because patients with rectal cancer usually survive for long periods, the incidence of the parastomal hernia may be even higher than reported.

Parastomal hernia can not only significantly reduce patients’ quality of life, but also cause a variety of specific complications such as pain, bleeding, bowel obstruction, and bowel strangulation [4, 5]. Although not all cases of parastomal hernia require surgical repair, there are a variety of surgical techniques. Local fascial repair, relocation of the stoma, and local repair of the parietal defect using meshes are the three basic methods of repair. The surgical approach may be open or laparoscopic. The treatment results were disappointing due to the 16–52 % recurrence rate and about 14–23 % other complications [6–9].

Because of the unsatisfactory results of existing treatments, it has been proposed that the best solution is to prevent parastomal hernia at the time of stoma formation. Some studies have found that prophylactic placement of mesh was associated with a significant reduction the occurrence of parastomal hernia without increasing morbidity [10], but this association was not found in other studies [11]. For this reason, a systematic review and meta-analysis of randomized controlled trials was performed to determine the efficacy and safety of prophylactic mesh in patients with rectal cancer after end-colostomy.

## Materials and methods

This systematic review and meta-analysis was conducted in accordance with Preferred Reporting Items for Systematic Reviews and Meta-analyses (PRISMA) [12].

### Literature search and inclusion criteria

Two authors (S.W. and B.Z.) independently searched electronic databases (inception to September 2015) including PubMed, Embase, and the Cochrane Library. Search terms included “rectal neoplasms,” “rectal cancer,” “colostomy,” “surgical mesh,” and “parastomal hernia,” and were searched in titles and abstracts. Free text searches and MeSH searches were performed. The search had no language restrictions. References of the articles included were also read to identify related articles.

Studies that met the following criteria were included: (1) population: patients receiving permanent end-colostomy surgery (Miles or Hartmann operation) to treat cancer of the rectum; (2) intervention: mesh insertion at the time of formation of end-colostomy; (3) comparison: no mesh insertion at the time of formation of end-colostomy; (4) outcome measure: the incidence of parastomal hernia; (5) study design: RCT.

### Data extraction, outcome measures, and assessment of risk of bias

Two authors independently extracted the data (S.W. and W.W.), and discrepancies were resolved through discussion. When no consensus could be reached, a third specialist was consulted (C.J.). The following information was extracted from each selected study: first author, year of publication, publishing country, published journal, type of mesh, position of mesh placement, location of stoma, and diagnostic bases of parastomal hernia. The primary outcome was the incidence of parastomal hernia. Secondary outcomes included stoma-related morbidity and reoperation related to parastomal hernia. Parastomal hernia was defined clinically and radiologically by a CT scan performed to identify possible subclinical parastomal hernia [13].

Assessment for risk of bias was conducted in accordance with the *Cochrane handbook for* *systematic reviews* *of* *interventions* (version 5.1.0) by two authors (S.W. and G.S.) [14]. Risk of bias was judged as “low risk,” “unclear risk,” and “high risk” according to the following domains: random sequence generation, allocation concealment, blinding of participants and personnel, blinding of outcome assessment, incomplete outcome data, selective reporting, and other bias.

The overall quality of the evidence for each outcome was evaluated using the Grading of Recommendations Assessment, Development and Evaluation (GRADE) approach [15]. GRADE Working Group grades of evidence were as follows: high quality: further research is very unlikely to change our confidence in the estimate of effect; moderate quality: further research is likely to have an important impact on our confidence in the estimate of effect and may change the estimate; low quality: further research is very likely to have an important impact on our confidence in the estimate of effect and is likely to change the estimate; and very low quality: any estimate of effect is very uncertain.

### Statistical analysis

Pooled RR and 95 % CI were estimated for each outcome. Statistical heterogeneity was assessed using χ^2^ and *I*^*2*^ statistics. Heterogeneity was considered significant if the *P* value (χ^2^) was <0.1 and *I*^*2*^ was >50 %. A random effects model was used regardless of heterogeneity. Whenever significant heterogeneity was present, possible sources of heterogeneity were assessed. For instance, a sensitivity analysis was performed and the study was excluded if the results were outside the range established by the others. Subgroup analysis was also performed, and the subgroups were based on the position of mesh placement (sublay vs. IPOM). The effects of treatment in different subgroups were compared using tests for interaction. The *P* value <0.05 supports a different treatment effect in the tested subgroups [16]. Potential publication bias was assessed by a visual inspection of the Begg's funnel plots where the log RR was plotted against their standard errors. The Begg’s test was used to measure the potential presence of publication bias [17]. Statistical analysis was performed with Stata 12.0 (Stata Corporation, College Station, TX, USA) and RevMan 5.3 (Nordic Cochrane Centre, Cochrane Collaboration, Copenhagen, Denmark).

## Results

### Search results and study characteristics

**Fig. 1:**
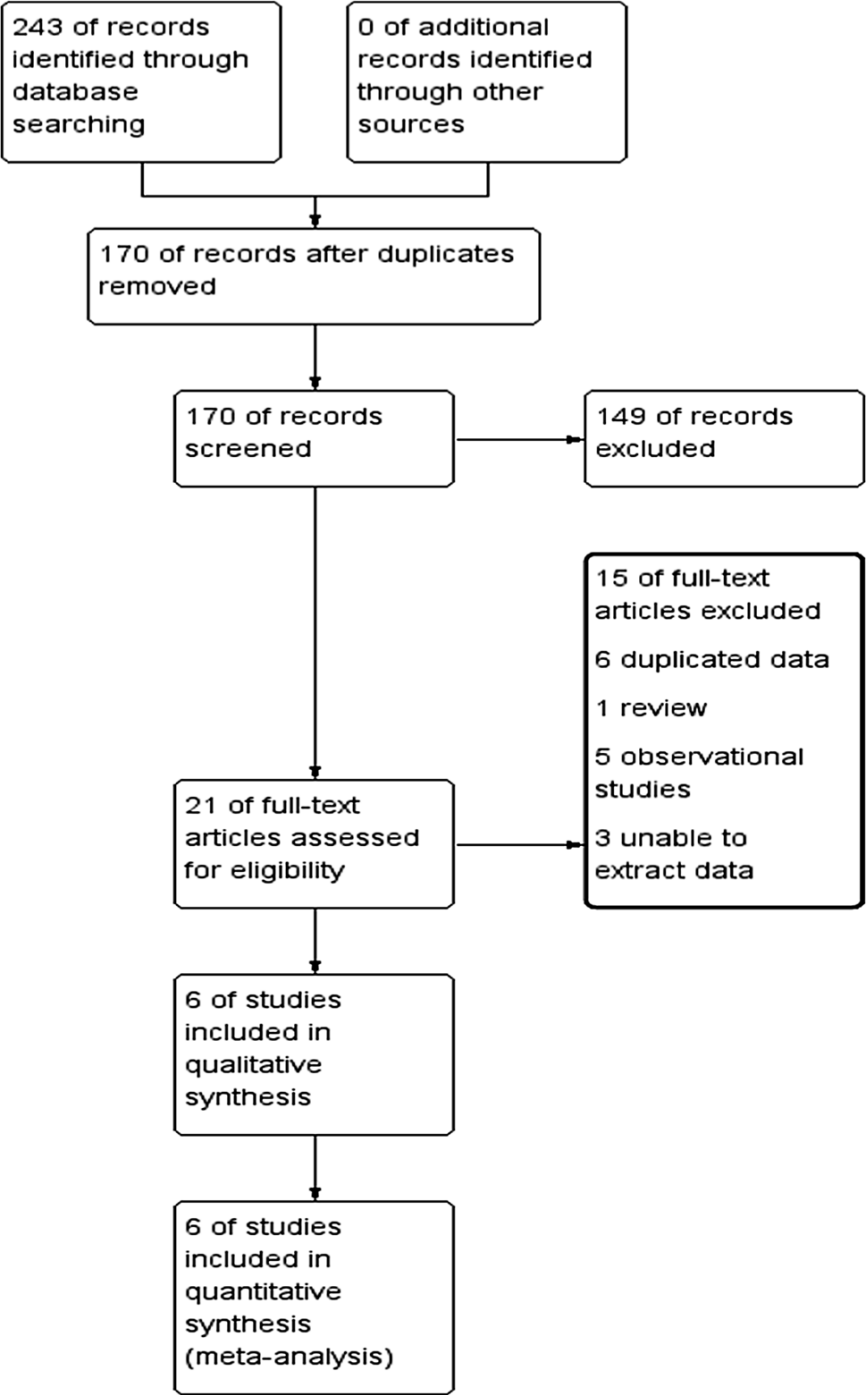
Flow chart of study screening and selection

The initial search identified 243 studies. After removal of 73 duplicate studies, 170 articles remained. After reading of the titles and abstracts, 149 studies were excluded due to irrelevant content, leaving 21 for full-text review. Upon further review, 15 more studies were excluded for the following reasons: six studies had duplicate data [18–23], one review [24], three studies with unavailable data [25–27], and five observational studies [11, 28–31]. Six articles were included [32–37] (Fig. 1).

All of the studies included here were published between 2009 and 2015. Six studies comprising 309 patients were included in the meta-analysis. This included 156 patients with mesh and 153 patients without mesh. Study sample size ranged from 36 to 70 patients. Among these six studies, all reported the incidence of parastomal hernia, five reported stoma-related morbidity [32–34, 36, 37], and four reported reoperation related to parastomal hernia [33, 34, 36, 37]. There were three studies of sublay placement [32, 34, 36] and three studies of intraperitoneal onlay mesh (IPOM) placement [33, 35, 37]. Synthetic mesh was used in the mesh group. Further characteristics of these studies are given in Tables 1 and 2.

**Table 1:** Details of the articles included

Reference	Year	Country	Journal	Type of mesh	Position of mesh placement	Location of stoma	Diagnostic bases on parastomal hernia
Lambrecht et al. [32]	2015	Norway	Colorectal Dis	Large-pore, low-weight polypropylene mesh	Sublay	Through the rectus abdominis muscle	Clinical examination
Lopez-Cano et al. [33]	2012	Spain	Hernia	Large-pore lightweight mesh made of polypropylene encapsulated with polydioxanone	IPOM	Through the rectus abdominis muscle	CT scan
Serra-Aracil et al. [34]	2009	Spain	Ann Surg	Ultrapro lightweight mesh	Sublay	Through the rectus abdominis muscle	Clinical examination and CT scan
Serra-Aracil et al. [35]	2015	Spain	Dis Colon Rectum	Large-pore lightweight composite mesh	IPOM	None	None
Tarcoveanu et al. [36]	2014	Romania	Chirurgia	Polypropylene mesh	Sublay	Through the rectus abdominis muscle	Clinical examination
Vierimaa et al. [37]	2015	Finland	Dis Colon Rectum	Dual-component structure composed of polyvinylidene fluoride and polypropylene	IPOM	Through the rectus abdominis muscle	Clinical examination and CT scan

Sublay denotes either behind the rectus muscle above the posterior rectus sheath or between posterior sheath and peritoneum

IPOM denotes intraperitoneal onlay mesh implantation and direct contact between mesh and abdominal viscera

**Table 2:** Characteristics of the included articles

Reference	Approach	Sample size	Age (year) mean ± SD	Female N (%)	BMI (kg/m^2^) mean ± SD	Follow-up duration, months median
Lambrecht et al. [32]	Mesh	32	64 ± 4.0	10 (31.3)	24.6 ± 0.6	36
	Non-mesh	26	63 ± 4.1	5 (19.2)	25.5 ± 0.8	48
Lopez-Cano et al. [33]	Mesh	19	72.2 ± 7.6	8 (42.1)	26.3 ± 3.2	10.6
	Non-mesh	17	65.9 ± 13.9	10 (58.8)	27.5 ± 4.7	10.6
Serra-Aracil et al. [34]	Mesh	27	67.5 ± 8.8	5 (18.5)	25.6 ± 2.9	29
	Non-mesh	27	67.2 ± 9.7	8 (29.6)	27.3 ± 3.5	29
Serra-Aracil et al. [35]	Mesh	23				18
	Non-mesh	26				18
Tarcoveanu et al. [36]	Mesh	20				20
	Non-mesh	22				20
Vierimaa et al. [37]	Mesh	35	67.1 ± 10.7	17 (48.6)	26.2 ± 4.6	12
	Non-mesh	35	65.1 ± 11.7	16 (45.7)	25.4 ± 4.3	12

*SD* Standard deviation, *BMI* body mass index

### Risk of bias and grades of evidence

**Fig. 2:**
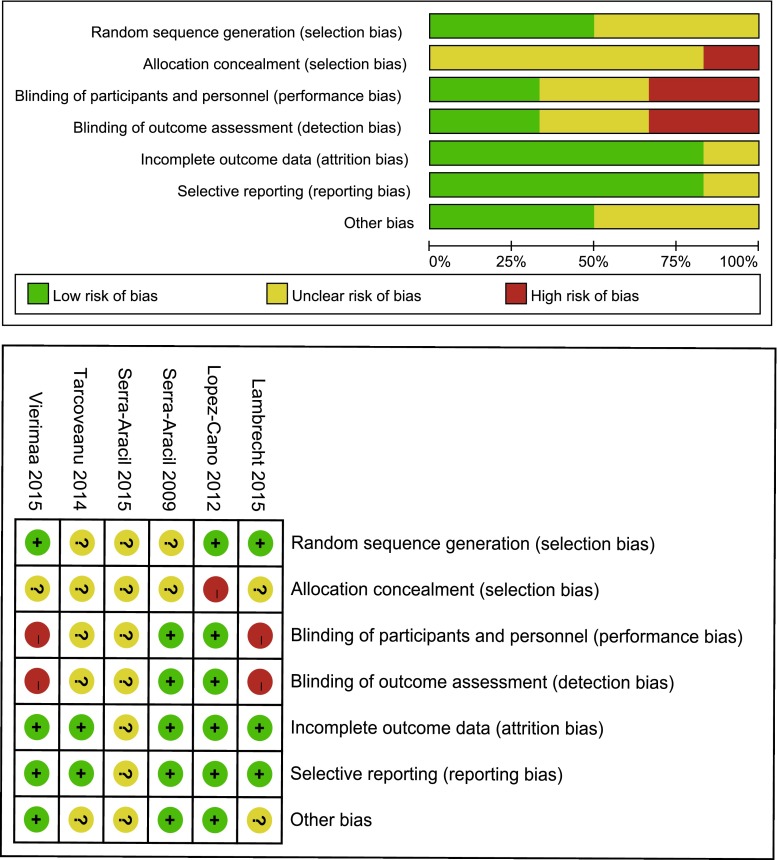
Assessment for risk of bias

Allocation concealment was inadequate in all studies. Outcome assessment was blinded in two studies. The details for risk of bias are presented in Fig. 2. GRADE Working Group grades of evidence were moderate for the incidence of parastomal hernia, low for stoma-related morbidity, and moderate for reoperation related to parastomal hernia.

### Primary outcome: incidence of parastomal hernia

**Fig. 3:**
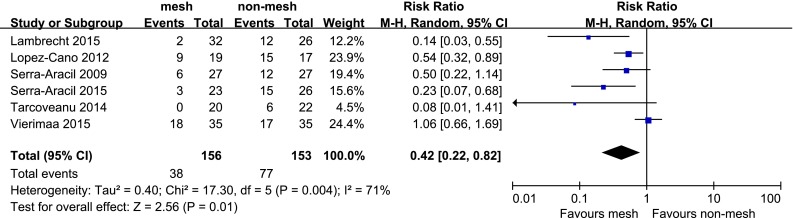
Forest plot of the incidence of parastomal hernia

All studies reported the incidence of parastomal hernia. The diagnosis of parastomal hernia was based solely on clinical examination in two studies [32, 36], solely on CT scans in one study [33], and on clinical examination and CT scans in two studies [34, 37]. One study did not disclose the diagnostic basis [35]. The incidences of parastomal hernia in the mesh group and non-mesh group were 24.4 and 50.3 %, respectively. The incidence of parastomal hernia was lower in the mesh group (RR, 0.42; 95 % CI 0.22–0.82, Fig. 3), and significant heterogeneity was observed among the studies (*P* = 0.004, *I*^*2*^ = 71 %).

### Sensitivity analysis

Significant heterogeneity was observed across all trials in the primary outcome (*P* = 0.004, *I*^*2*^ = 71 %). As shown in Fig. 3, the results of the study conducted by Vierimaa et al. [37] were outside of the range of the others and probably contributed to the heterogeneity. After excluding this study, the results showed the mesh group to have a lower incidence of parastomal hernia than the non-mesh group (RR, 0.34; 95 % CI 0.18–0.64). No significant heterogeneity was observed among the remaining studies (*P* = 0.11, *I*^*2*^ = 47 %).

### Subgroup analysis

There was no significant difference between studies of sublay placement (three trials, RR, 0.25; 95 % CI 0.08–0.80) and studies of IPOM placement (three trials, RR, 0.57; 95 % CI 0.27–1.21) by the test of interaction (*P* = 0.24, *I*^*2*^ = 28.9 %).

### Effect of missing data

Of the six included studies, two did not report the number of deaths and missing visits [35, 36], two studies used intention-to-treat analysis [34, 37], and the remaining studies reported the number of deaths and missed visits but did not use intention-to-treat analysis [32, 33]. Assuming that eleven people who died in the mesh group developed a parastomal hernia, the incidence of parastomal hernia remained lower in the mesh group (31.4 %) than in the non-mesh group (50.3 %) (RR, 0.61; 95 % CI 0.39–0.96; *P* = 0.03).

### Secondary outcomes

**Fig. 4:**
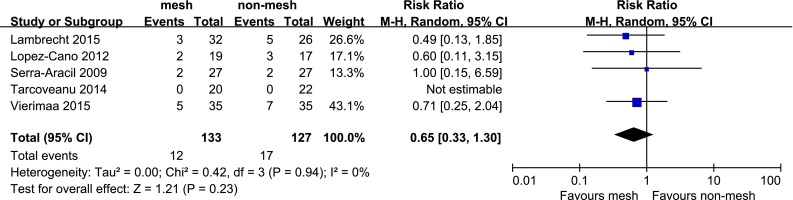
Forest plot of stoma-related morbidity

Five studies reported stoma-related morbidity and there was no heterogeneity among them (*P* = 0.94, *I*^*2*^ = 0 %). In the random effects model, stoma-related morbidity was similar between the mesh and non-mesh groups (RR, 0.65; 95 % CI 0.33–1.30) (Fig. 4).

**Fig. 5:**
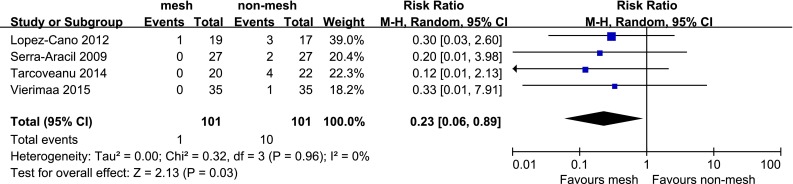
Forest plot of reoperation related to parastomal hernia

Four studies reported, reoperation related to parastomal hernia. The pooled results from these studies showed the mesh group to be associated with a lower risk of reoperation related to parastomal hernia (RR, 0.23; 95 % CI 0.06–0.89, Fig. 5), with no heterogeneity among the studies (*P* = 0.96, *I*^*2*^ = 0 %).

### Publication bias

Assessment of publication bias showed that there was no potential publication bias among the included studies (Begg’s test, *P* = 0.13).

## Discussion

Our study evaluated efficacy of prophylactic mesh at end-colostomy construction. The results revealed that, for sigmoid end-colostomy, prophylactic placement of a mesh reduced the incidence of parastomal hernia, reoperation related to parastomal hernia, and made no significant difference in terms of stoma-related morbidity.

These results were similar to those published in a previous meta-analysis by Wijeyekoon et al. [38]. However, the selection of the study population was different between these two studies. The prior study included all patients with end and loop stomas. Because the incidence of parastomal hernia was different between end and loop stomas [39, 40], the present study only included those patients who underwent permanent end-colostomy surgery to treat cancer of the rectum. The prior analysis only included three articles involving a total of 129 patients. The present meta-analysis included more recently published articles and a greater number of patients than Wijeyekoon et al. did; therefore, it was more likely to detect differences.

Parastomal hernia is caused by enlargement of the trephine opening in the abdominal wall, which is usually due to the working of mechanical tangential stress on the circumference of the opening. Reinforcing the abdominal wall with mesh during the initial operation might reduce the incidence of parastomal hernia. The mesh group was found to have a distinct advantage over the non-mesh group with respect to the incidence of the parastomal hernia. In the studies included here, mesh was placed as a sublay or in the intraperitoneal plane. The sublay technique is considered feasible and can lower the incidence of parastomal hernia [10]. The intraperitoneal technique includes the modified Sugarbaker method, the keyhole technique, and the sandwich technique. The keyhole technique involves cutting a hole in the mesh to destroy its architecture and reduce its tensile strength, resulting in gradual widening of the opening. The modified Sugarbaker technique is believed to be superior to the keyhole technique because it is associated with fewer recurrences [41, 42]. Although the keyhole technique was used on more patients than the modified Sugarbaker technique in the included studies, the incidence of parastomal hernia was lower in mesh group than that in non-mesh group.

What concerns surgeons is the risk of complications attributable to insertion of a prosthetic material into a potentially contaminated field such as an open bowel. They are particularly concerned about the risk of wound infection, which may necessitate mesh removal. The intraperitoneal technique is an aseptic procedure involving no contact between the mesh and the broken ends of the transected colon. The sigmoid colon used to create the stoma is passed through the abdominal wall before the mesh is placed. In this meta-analysis, there were no cases of mesh removal for the reason of infection in cases treated with the sublay technique. This may be because of the lower rate of inflammatory reaction and better defense against infection with large-pore lightweight mesh [43]. After preparation of the bowel and the management of pollution, mesh can be used in the presence of open bowel with minimal risk of infection [44, 45]. Although Aldridge [46] reported a case of erosion and perforation of colon by synthetic mesh, this complication is rare, and improvements to materials and techniques have made it rarer. In the studies examined here, stoma detachment and necrosis of the colostomy were more common than erosion or perforation, and it was not the mesh but the blood supply and tension of sigmoid colon that cause these complications. In this way, no significant differences in stoma-related complications were found between the two groups.

Analysis showed the mesh group to have advantages not only related to the incidence of the parastomal hernia but also related to reoperation for parastomal hernia. The significant increases in aperture size were observed in the non-mesh group after CT scan [32], which can cause discomfort, may go some way toward explaining this.

Even though the results are encouraging, there are still factors that must be considered. Some of the patients in the mesh group died from recurrence of cancer within 12 months. Even when it was assumed that everyone in the mesh group who died had developed a parastomal hernia, the results of the meta-analysis did not change. It is not appropriate to place a mesh in patients whose survival time is less than 1 year. Once these patients are excluded, prophylactic mesh placement can be considered cost-effective. The incremental cost-effectiveness ratio (ICER) was $6676/QALY (quality adjusted life years) [47]. Several studies have found female gender, higher body mass index, and lower levels of preoperative albumin to offer significant independent risk for parastomal hernia [48, 49]. However, none of the studies evaluated the placement of mesh in these specific patients. This may be a suitable subject for future studies.

This study has a number of limitations that should be considered. First, there are different protocols for diagnosis of parastomal hernia. Parastomal hernia, whether diagnosed by clinical examination or by CT scan, has no consistent definition. Clinical examination is a subjective method, and it can be especially difficult to identify parastomal hernia in obese patients. Although CT scanning is an objective method, it is subject to false negatives. A definition of parastomal hernia for use with clinical examination that matches findings from CT scans should be established [50]. Second, the follow-up period was different in different studies, though it was more than 12 months in most of them. Although most cases of parastomal hernia appear within the first 12 months of construction, a higher incidence of parastomal hernia is reported with longer follow-up [19, 25]. Future trials should have follow-up times of 12 months or longer. Finally, although we included RCT, some of the following factors might still produce a biased estimate of the effects of treatment effect. Random sequence generation was adequate in only three of the studies included here. Only one study provided sufficient information to assess allocation concealment, and it did so inadequately. Blinding of outcome assessment was used in two of the included studies.

In conclusion, available evidence suggests that prophylactic mesh at end-colostomy construction after surgery for low rectal cancer is an effective and safe procedure. However, the results should be interpreted with caution because of the heterogeneity among the studies and prophylactic mesh placement in all patients or in patients with specific needs requires further study.
